# Clinical characteristics of patients hospitalized for COVID-19: comparison between different age groups

**DOI:** 10.1186/s12877-023-04626-2

**Published:** 2024-01-11

**Authors:** Ginevra Fabiani, Carolina Cogozzo, Anna De Paris, Valentina Di Maria, Alessia Lagomarsini, Olimpia Masotti, Simona Matteini, Elisa Paolucci, Lorenzo Pelagatti, Francesco Pepe, Maurizio Villanti, Francesca Todde, Riccardo Pini, Francesca Innocenti

**Affiliations:** High-Dependency Unit, Department of Clinical and Experimental Medicine, Careggi University Hospital, Lg. Brambilla 3, 50134 Florence, Italy

**Keywords:** COVID-19, Prognostic scores, Mortality rate, Respiratory failure

## Abstract

**Background:**

To test whether known prognosticators of COVID-19 maintained their stratification ability across age groups.

**Methods:**

We performed a retrospective study. We included all patients (*n* = 2225), who presented to the Emergency Department of the Careggi University Hospital for COVID-19 in the period February 2020—May 2021, and were admitted to the hospital. The following parameters were analyzed as dichotomized: 1) SpO_2_/FiO_2_ ≤ or > 214; 2) creatinine < or ≥ 1.1 mg/dL; 3) Lactic dehydrogenase (LDH) < or ≥ 250 U/mL; 4) C Reactive Protein (CRP) < or ≥ 60 mg/100 mL. We divided the study population in four subgroups, based on the quartiles of distribution of age (G1 18–57 years, G2 57–71 years, G3 72–81 years, G4 > 82). The primary end-point was in-hospital mortality.

**Results:**

By the univariate analysis, the aforementioned dichotomized variables demonstrated a significant association with in-hospital mortality in all subgroups. We introduced them in a multivariate model: in G1 SpO2/FiO2 ≤ 214 (Relative Risk, RR 15.66; 95%CI 3.98–61,74), in G2 creatinine ≥ 1.1 mg/L (RR 2.87, 95%CI 1.30–6.32) and LDH ≥ 250 UI/L (RR 8.71, 95%CI 1,15–65,70), in G3 creatinine ≥ 1.1 mg/L (RR 1.98, 95%CI 1,17–3.36) and CRP ≥ 60 ng/L (RR 2.14, 95%CI 1.23–3.71), in G4 SpO_2_/FiO_2_ ≤ 214 (RR 5.15, 95%CI 2.35–11.29), creatinine ≥ 1.1 mg/L (RR 1.75, 95%CI 1.09–2.80) and CRP ≥ 60 ng/L (RR 1.82, 95%CI 1.11–2.98) were independently associated with an increased in-hospital mortality.

**Conclusions:**

A mild to moderate respiratory failure showed an independent association with an increased mortality rate only in youngest and oldest patients, while kidney disease maintained a prognostic role regardless of age.

## Translational significance

The study compares clinical characteristics of patients with COVID-19 in different age groups, in order to find an explanation for the excess mortality among elderly patients. Upon the presentation to the Emergency Department, patients aged ≥ 65 years had a slightly worse respiratory failure, even though in the range of a mild to moderate impairment, as well as a more pronounced derangement of inflammatory biomarkers, compared to their younger counterpart. Age-related changes in lung structure and function as well as the reduced effectiveness of the immune system could justify the unfavorable outcome of elderly patients and underline the need for an appropriate disposition for these patients.

## Background

The SARS-CoV-2 infection caused the first pandemic induced by a coronavirus and the fifth documented pandemic in the recent history [1, 2]. The infection by COVID-19 may induce a wide range of clinical pictures, from an asymptomatic infection to a severe pneumonia with concomitant multi-organ failure. The immune asset of hosts, as well as previous medical conditions, plays a relevant role in determining the response to the infection and the outcome. Among demographic factors, an advanced age was identified as one of the strongest risk factors for short-term mortality in patients with COVID-19 [3]. Few papers compared the early clinical characteristics among patients of different age groups, in order to ascertain to what extent, the initial clinical picture was different based on patients’ age [4].

On the other side, different prognosticators have been identified by several authors, but they have not been specifically tested in population of different ages [5, 6]. The disproportionally high mortality of elderly people requires a test of their prognostic stratification ability in this population. Prognosticators included both indices of inflammatory activation and parameters of organ damage [7]. Among the formers, C-reactive protein (CRP), an acute-phase protein synthesized by the liver in response to interleukin-6 (IL-6), is widely available in the clinical practice and several authors reported an association between high CRP concentrations and a more severe disease [8, 9]. Serum lactate dehydrogenase (LDH) levels were often elevated in patients with severe COVID-19, reflecting the extensive pulmonary damage [10]. The presence of elevated serum lactic dehydrogenase (LDH) upon the initial presentation has been shown to be associated to the progression to respiratory failure and death. Therefore, the LDH level was regarded as a useful indicator for the early identification of patients at risk of an unfavorable outcome. The presence of a known renal failure, even mild, was associated with an adverse prognosis.

The aim of this research was to analyze the clinical characteristics of patients with COVID-19 in patients of different age groups and to verify whether known prognosticators maintained their stratification ability regardless of patients’ age.

## Methods

### Patients selection

We performed a retrospective analysis of the medical records of all patients, who accessed the ED of the Careggi University Hospital in the period between 24 February 2020 and 31 May 2021, with SARS-CoV-2 infection. They were identified from medical records, based on the following criteria: age ≥ 18 years, nasopharyngeal swab positive for SARS-CoV2 and need for hospitalization. The ethics committee and institutional review board approved this study (NO. 17,104). The clinical parameters of the selected patients were included in a corporate database, from which the data used for the present study were extracted.

Upon admission to the ED, the following data were collected for each patient:

- main anamnestic data, with particular attention to previous medical conditions and presenting symptoms
- vital signs (body temperature, heart rate, respiratory rate, blood pressure, Glasgow Coma Scale).
- arterial blood gas data
- routine blood tests.

We selected four parameters, which were significantly different between all groups (SpO_2_/FiO_2_, Creatinine, PCR, LDH) and represented different aspects of the disease, namely the inflammatory activation and the severity of organ damage. We decided to evaluate the respiratory function by mean of SpO_2_/FiO_2_ ratio, in order to include even patients with mild respiratory failure, for whom, in the ED, it may not have been deemed necessary to perform an arterial blood gas. The ratio SpO_2_/FiO_2_ was categorized according to the study by Pandharipande and coll [11].

From the arterial blood gas, which was available in 1729 patients (78%), we derived parameters to calculate PaO_2_/FiO_2_ ratio.

The following parameters were analyzed both as continuous values and dichotomized: 1) SpO_2_/FiO_2_ ≤ or > 214, corresponding to a SOFA score of 2.00 [11]; 2) creatinine < or ≥ 1.1 mg/L; 3) LDH < or ≥ 250 UI/L; 4) CRP < or ≥ 60 ng/L. Creatinine was dichotomized based on the mean value of the present study population. As far as we considered available papers, we found that the prognostic value of LDH was tested considering its conventional normal value. On the other side, all authors reported that elevated levels of CRP were associated with an adverse prognosis, but the cut-off value, mostly derived from the median value of single papers, showed high variability (from 80 to 220 ng/L). Therefore, we decided to dichotomize the value based on our median value (= 59 ng/L). Patients with SpO_2_/FiO_2_ > 214 were diagnosed with mild to moderate respiratory failure.

We divided the study population in four subgroups based on the quartiles of distribution of age: group 1 (G1, *n* = 554), aged 18–57 years, group 2 (G2, *n* = 520), aged 57–71 years, group 3 (G3, *n* = 558), aged 72–81 years, and group 4 (G4, *n* = 593), aged 82 and more. The primary end-point was the in-hospital mortality.

### Statistical analysis

Dichotomous data were reported as absolute number and percentage. Continuous parameters were reported as mean ± standard deviation or median and interquartile range, based on their distribution. For continuous variables, the null hypothesis was tested using Repeated Measures Analysis for data with normally distributed data; the Bonferroni test was used for the post-hoc analysis. The dichotomous variables were analyzed through the construction of the contingency tables and the execution of the χ2 test; in the case of comparison between more than two groups, the Bonferroni correction was applied. To test the independent association of the selected prognosticators with the in-hospital mortality, we performed a univariate regression analysis and, thereafter, we introduced all variables in a multivariate analysis.

Analyses were performed using the SPSS software, version 27 (SPSS Statistics, IBM Corporation, Chicago, Ill, USA).

## Results

Between 24 February 2020 and 31 May 2021, 2435 patients affected by COVID-19 presented to the ED of the Careggi University Hospital; among them, 2225 were hospitalized and represent our study population. The mean age was 68 ± 16 years, 58% males. The anamnestic characteristics and the presenting symptoms of COVID-19, the vital parameters and the data from the arterial blood gas are respectively reported in Tables 1 and 2, in the whole population and in subgroups based on age quartiles. As expected, all comorbidities were more frequent among the older than the younger patients, with an overall low prevalence of previous medical conditions; only arterial hypertension affected 51% of the study population. Dyspnea was the most frequent presenting symptom in all age groups, with a similar prevalence, followed by cough, which was significantly more frequent among G1 than among the other subgroups. Compared to older patients, the younger groups reported more frequently gastrointestinal symptoms, while syncope was less frequently the presenting symptom.

**Table 1:** Anamnestic data in the whole population and in subgroups based on age quartiles

	All patients **(*n* = 2225)**	**G1** **(*n* = 55**8**)**	**G2** **(*n* = 52**2**)**	**G3** **(*n* = 55**9**)**	**G4** **(*n* = 58**6**)**
Age (years)	68 ± 16				
Male gender (%)	1295 (58%)	367 (66%)	323 (62%)	318 (57%)	287 (49%)
Hypertension (%)	1110 (51%)	117 (22%) *°§	249 (49%) #◊	345 (63%) **●**	399 (69%)
COPD (%)	187 (9%)	3 (1%) *°§	28 (6%) #◊	75 (14%)	81 (14%)
Asthma (%)	89 (4%)	32 (6%) *°§	23 (5%)	20 (4%)	14 (2%)
DM (%)	391 (18%)	47 (9%) *°§	95 (19%) #	132 (24%)	117 (20%)
CAD (%)	290 (13%)	8 (2%) *°§	51 (10%) #◊	101 (19%)	130 (22%)
CHF (%)	165 (8%)	4 (1%) *°§	16 (3%) #◊	48 (9%) **●**	97 (17%)
AF (%)	151 (7%)	1 (0.2%) *°§	10 (2%) #◊	39 (8%) **●**	101 (20%)
Cirrhosis (%)	54 (3%)	11 (2%)	14 (3%)	17 (3%)	12 (2%)
**Presentation symptoms**					
Cough (%)	485 (27%)	170 (40%)	115 (29%) ◊	108 (24%)	92 (17%)*°§
Dyspnea (%)	717 (42%)	163 (40%)	158 (43%)	186 (43%)	210 (43%)
Myalgia (%)	7 (0.3%)	1 (0.2%)	4 (0.9%)	2 (0.4%)	0
Fatigue (%)	234 (12%)	68 (14%)	60 (13%)	43 (9%)	63 (12%)
Nausea (%)	2 (0.1%)	0	1 (0.2%)	1 (0.2%)	0
Vomitus (%)	66 (3%)	24 (5%) *	6 (1%)	15 (3%)	21 (4%)
Diarrhea (%)	105 (5%)	38 (8%) *°	15 (3%)	22 (4%)	29 (5%)
Syncope (%)	74 (4%)	9 (2%) §	14 (3%) ◊	17 (3%) **●**	34 (6%)

*COPD* Chronic Obstructive Pulmonary Disease, *DM* Diabetes mellitus, *CAD* Coronary Artery Disease, *CHF* Congestive Heart Failure, *AF* Atrial fibrillation

^*^p < 0.05 G1 vs G2; ° *p* < 0.05 G1 vs G3; § *p* < 0.05 G1 vs G4; # *p* < 0.05 G2 vs G3; ◊ *p* < 0.05 G2 vs G4; ● *p* < 0.05 G3 vs G4

**Table 2:** Vitals and arterial blood gas parameters in the whole population and subgroups based on age quartiles

	**All** **(*n* = 2225)**	G1 (*n* = 558)	G2 (*n* = 522)	G3 (*n* = 559)	G4 (*n* = 586)
HR (b/min)	87 ± 17	93 ± 16 *°§	87 ± 15	84 ± 16	86 ± 20
SBP (mmHg)	133 ± 21	130 ± 16 *°§	133 ± 19	134 ± 22	134 ± 24
DBP (mmHg)	76 ± 12	79 ± 11**°§**	78 ± 12 **#◊**	75 ± 12	73 ± 13
RR (a/min)	20 ± 6	20 ± 6 **§**	20 ± 6	21 ± 6	21 ± 6
pSO_2_ (%)	92 ± 7	93 ± 5**°§**	92 ± 5	92 ± 8	92 ± 7
FiO_2_ (%)	29 ± 20	25 ± 15 §	27 ± 20 **◊**	28 ± 18 **●**	35 ± 26
GCS	15 [15–15]	15 [15–15]** °§**	15 [15–15]** #◊**	15 [15–15]** ●**	15 [15–15]
BT (°C)	37 ± 1	37 ± 1**°§**	37 ± 1 **◊**	37 ± 1	37 ± 1
pH	7.46 ± 0.06	7.46 ± 0.04 **§**	7.47 ± 0.05 **#◊**	7.45 ± 0.6	7.45 ± 0.06
pO_2_ (mmHg)	68 ± 27	70 ± 24	65 ± 21 **◊**	67 ± 25 **●**	72 ± 36
pCO_2_ (mmHg)	36 ± 8	36 ± 7	35 ± 6 **◊**	36 ± 8	36 ± 9
HCO_3_^−^ (mEq/L)	25 ± 4	26 ± 3	26 ± 4 **◊**	25 ± 4	25 ± 5
LAC (mEq/L)	1.3 ± 1.2	1.0 ± 0.7**°§**	1.2 ± 1.0 **#◊**	1.5 ± 1.6	1.5 ± 1.3
P/F	282 ± 163	305 ± 81*°§	278 ± 77◊	276 ± 85●	256 ± 101
SO_2_/FiO_2_	391 ± 102	411 ± 93°§	395 ± 97◊	389 ± 99●	368 ± 114

*HR* Heart rate, *SBP* Systolic Blood Pressure, *DBP* Diastolic Blood Pressure, *RR* Respiratory Rate, *GCS* Glasgow Coma Scale, *BT* Body Temperature, LAC Lactate

^*^ *p* < 0.05 G1 vs G2; ° *p* < 0.05 G1 vs G3; § *p* < 0.05 G1 vs G4; # *p* < 0.05 G2 vs G3; ◊ *p* < 0.05 G2 vs G4; ● *p* < 0.05 G3 vs G4

Compared to their younger counterpart, older patients showed a lower heart rate and slightly higher blood pressure, mild tachypnea with hypoxemia. Despite these differences, near all patients, regardless of their age, were hemodynamically stable, with mild to moderate hypoxemic respiratory failure. Older patients showed a worse renal function and higher levels of inflammation markers than younger subjects (Table 3). Further differences were also observed, mainly in the total and differential white blood cell count, but with all values within the normal range. For the most part of the examined parameters, G1 showed better values than other subgroups, G2 was in an intermediate position, while we found marginal differences between G3 and G4.

**Table 3:** Laboratory parameters in the whole population and in subgroups based on age quartiles

	**All** **(*n* = 2225)**	G1 (*n* = 558)	G2 (*n* = 522)	G3 (*n* = 559)	G4 (*n* = 586)
ALT (U/L)	38 ± 114	47 ± 53°**§**	41 ± 54	30 ± 27	27 ± 34
Creatinine (mg/dL)	1.17 ± 1.01	1.00 ± 1.16**°§**	1.06 ± 0.75 **#◊**	1.24 ± 1.06	1.37 ± 0.95
WBC (Ux ${10}^{9}$/L)	8.92 ± 35.36	7.59 ± 4.70§	7.62 ± 4.71**◊**	8.10 ± 6.11	8.69 ± 5.62
RBC (Ux ${10}^{6}/\mathbf{L}$)	4.81 ± 10.39	4.92 ± 0.60*°**§**	4.73 ± 0.67**#◊**	4.51 ± 0.87●	4.22 ± 0.75
Hb (g/L)	13.4 ± 2.3	14.1 ± 1.7**°§**	14.0 ± 2.8 **#◊**	13.1 ± 2.1 **●**	12.4 ± 2.0
PLT (Ux ${10}^{6}/\mathbf{L}$)	217 ± 104	221 ± 86	215 ± 91	211 ± 100	221 ± 130
Neutrophils (Ux ${10}^{9}$/L)	76 ± 26	74 ± 14**°**	74 ± 18 **#**	79 ± 36	78 ± 31
Lymphocytes (Ux ${10}^{9}$/L)	15 ± 13	17 ± 11**°§**	16 ± 16 **◊**	14 ± 11	14 ± 12
N/L ratio	10.7 ± 22.9	8.7 ± 18.5°	8.9 ± 14.0#	13.0 ± 35.6	12.0 ± 16.5
K + (mEq/L)	4.2 ± 0.6	4.1 ± 0.5 *°	4.2 ± 0.5 **#**	4.3 ± 0.6	4.2 ± 0.7
Na + (mEq/L)	138 ± 7	137 ± 5 **§**	137 ± 4 **◊**	137 ± 5 **●**	139 ± 10
Glycemia (mg/dL)	138 ± 65	127 ± 57 *°	145 ± 83	141 ± 62	140 ± 56
Fibrinogen (mg/dL)	607 ± 226	616 ± 206	635 ± 297 **◊**	602 ± 191	573 ± 189
INR	1.54 ± 10.83	1.17 ± 0.25	1.20 ± 0.51	1.30 ± 0.64	2.46 ± 21.39
CRP (mg/100mL)	78 ± 70	64 ± 63 *°§	77 ± 71 **◊**	80 ± 69	91 ± 75
LDH (mU/mL)	356 ± 197	326 ± 128**°§**	365 ± 185	366 ± 2216	368 ± 230
Bilirubin (mg/dL)	0.55 ± 0.39	0.49 ± 0.29 *§	0.57 ± 0.42	0.55 ± 0.41	0.59 ± 0.42
IL6 (pg/mL)	175 ± 675	63 ± 160 **§**	174 ± 734	140 ± 514	300 ± 945

*ALT* Alanine aminotransferase, *WBC* White Blood Cells, *RBC* Red Blood Cells, *Hb* Hemoglobine, *PLT* Platelets, *HTC* Hematocrit, *CRP* C Reactive Protein

^*^ *p* < 0.05 G1 vs G2; ° *p* < 0.05 G1 vs G3; § *p* < 0.05 G1 vs G4; # *p* < 0.05 G2 vs G3; ◊ *p* < 0.05 G2 vs G4; ● *p* < 0.05 G3 vs G4

**Fig. 1:**
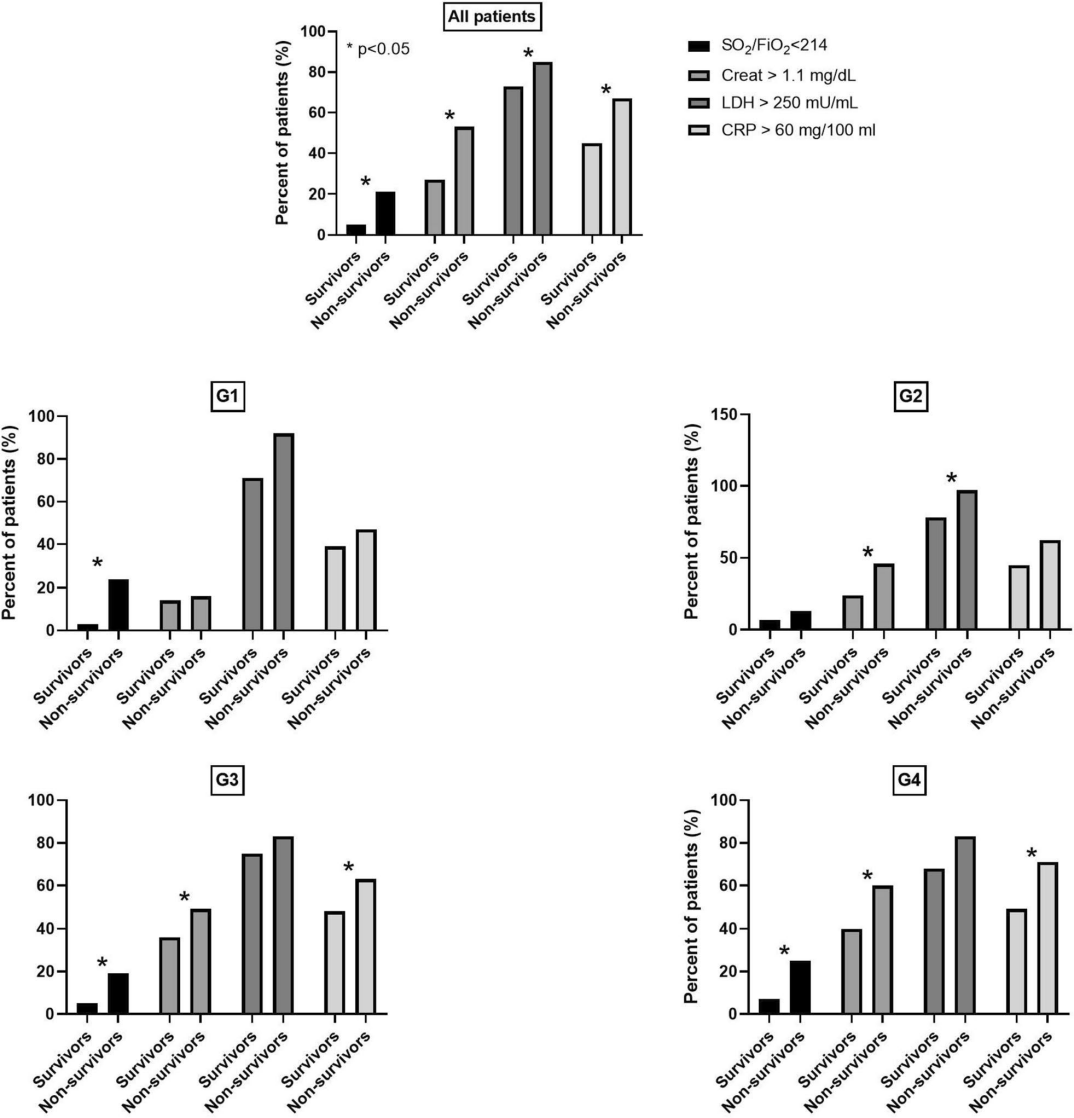
Percentage of survivors and non-survivors, who presented the pathological values of four dichotomized value (SpO_2_/FiO_2_, Creatinine, CRP and LDH) in the whole population and in different age groups

The mortality rate significantly increased with increasing age (G1 4%, G2 9%, G3 22% and G4 39%, *p* < 0.01 for all comparisons). In Fig. 1, we reported the percentage of survivors and non-survivors, who presented the pathological values of the parameters, which were evaluated as dichotomous variables, SpO_2_/FiO_2_ ≤ or > 214, creatinine < or ≥ 1.1 mg/L, LDH < or ≥ 250 UI/L and CRP < or ≥ 60 ng/L. In the whole population, non-survivors showed more frequently the worse value for all the variables. In the analysis in single subgroups, we confirmed that non-survivors showed always a more compromised respiratory function, but the higher value of inflammatory biomarkers was more frequent among non-survivors than among survivors only in patients aged > 70. In G1, 92 subjects were younger than 37 years and 22 patients died, all over 38 years of age. Among them, 5 patients (23%) showed SpO_2_/FiO_2_ ≤ 214, compared to 14 out of 536 survivors (3%, *p* = 0.005), all over 40 years of age. Therefore, G1 patients with unfavorable clinical parameters were at least in their forties. Despite the fact that we are dealing with very low numbers, they determined a significant difference between survivors and non-survivors.

The univariate analysis confirmed these results (Table 4). The multivariate analysis, which included all the aforementioned parameters, was performed for the whole study population and for single subgroups (Table 4). In the whole population, all the tested parameters demonstrated an independent association with an increased in-hospital mortality. In subgroups, a mild to moderate respiratory deterioration upon ED admission was an independent prognosticator only in youngest and oldest patients, while an impaired renal function confirmed its relevant prognostic value in all subgroups except the youngest subjects. On the other side, increased levels LDH and CPR showed a variable association with an adverse prognosis across age groups.

**Table 4:** Univariate association of selected clinical characteristics with all-cause mortality in all patients and different age groups

	Univariate analysis			Multivariate analysis		
	RR	95%CI	p	RR	95%CI	p
All (*n* = 2225)						
SO_2_/FiO_2_ < 214	4.99	3.59–6.94	< 0.001	3.40	2.23–5.19	< 0.001
Creatinine > 1.1 mg/L	3.05	2.44–3.82	< 0.001	2.92	2.18–3.90	< 0.001
LDH > 250 UI/L	2.06	1.46–2.91	< 0.001		-	
CPR > 60 UI/L	2.43	1.92–3.08	< 0.001	2.06	1.52–2.80	< 0.001
G1(*n* = 55**8**)						
SO_2_/FiO_2_ < 214	9.84	3.16–30.67	< 0.001	15.66	3.98–61,74	< 0.001
Creatinine > 1.1 mg/L	1.17	0.33–4.12	0.808		-	
LDH > 250 UI/L	4.92	0.63–38.33	0.128		-	
CPR > 60 UI/L	1.38	0.55–3.47	0.488		-	
G2 (*n* = 52**2**)						
SO_2_/FiO_2_ < 214	1.94	0.71–5.33	0.198		-	
Creatinine > 1.1 mg/L	2.58	1.37–4.85	0.003	2.87	1.30–6.32	0.009
LDH > 250 UI/L	10.35	1.39–78.86	0.022	8.71	1,15–65-70	0.036
CPR > 60 UI/L	1.96	1.02–3.75	0.044		-	
G3 *(n* = 55**9**)						
SO_2_/FiO_2_ < 214	4.62	2.40–8.89	< 0.001		-	
Creatinine > 1.1 mg/L	1.75	1.16–2.65	0.008	1.98	1,17–3.36	0.011
LDH > 250 UI/L	1.63	0.86–3.08	0.134		-	
CPR > 60 UI/L	1.82	1.18–2.79	0.007	2.14	1.23–3.71	0.007
G4 (*n* = 58**6**)						
SO_2_/FiO_2_ < 214	4.65	2.70–8.03	< 0.001	5.15	2.35–11.29	< 0.001
Creatinine > 1.1 mg/L	2.27	1.60–3.23	< 0.001	1.75	1.09–2.80	0.020
LDH > 250 UI/L	2.21	1.33–3.68	0.002		-	
CPR > 60 UI/L	2.59	1.78–3.77	< 0.001	1.82	1.11–2.98	0.017

*RR* Relative Risk, *CI* Confidence Interval, *SO*_*2*_*/FiO*_*2*_ Oxygen saturation/Fraction of inspired oxygen, *LDH* Lactic dehydrogenase, *CPR* C Reactive Protein

## Discussion

In a large population of patients admitted to the hospital for COVID-19, we compared the presenting characteristics and their respective prognostic value in different age groups. We confirmed a tenfold in-hospital mortality rate among patients aged over 82 years, compared to those under 52 years, with slightly different prognosticators. In fact, the presence of a mild to moderate respiratory impairment was associated with an adverse prognosis in youngest and oldest patients, while reduced renal function maintained a prognostic value across all groups except subjects aged < 50 years. An increased CRP level was independently associated with an unfavorable prognosis only in the elderly.

The initial clinical picture was characterized, as expected, by a higher prevalence of comorbidities among the elderly than among patients aged < 50 years, in the presence of an overall low burden of previous medical conditions. Among the symptoms, dyspnea was most frequently related to an unfavorable evolution and showed a similar prevalence in all age groups, while syncope occurred more frequently in aged than in young people. In terms of vitals and arterial blood gas parameters, compared to younger patients, older people showed a slightly greater impairment, with a mild to moderate respiratory failure and stable hemodynamics. As expected, laboratory parameters showed a more marked renal impairment in the elderly than in young patients, with a more pronounced alteration of inflammation markers in the former group. These differences were statistically significant, but their clinical relevance was limited. In fact, if we consider the classification of organ damage based on the SOFA score, the difference between the worst and the best value, both for the pulmonary and renal function, was 1 point.

Several studies confirmed the positive association between an advanced age and an increased fatality rate during COVID-19 [12, 13], with a multifactorial etiology. All the previous medical conditions, which had been proven to be associated with an unfavorable prognosis, were more frequent in the elderly than among their younger counterpart, including hypertension, diabetes, chronic obstructive pulmonary disease, chronic kidney disease and coronary artery disease [14]. The clinical presentation was similar between different age groups, in disagreement with the previous study, performed at the beginning of the pandemic. Martin-Sanchez and coll. [4] found significant differences between patients of different ages and hypothesized a role of the atypical presentation and late diagnosis for the excess mortality observed in the elderly. Perhaps due to a long experience with this kind of patients with the progression of the pandemic, we did not confirm this hypothesis and we evidenced that symptoms with a recognized prognostic weight had a similar prevalence across age groups. Only syncope showed a very low prevalence, although higher among aged than young patients. To the best of our knowledge, few papers compared the characteristics of COVID-19 across different age groups, and those few studies included only patients in the first wave of the pandemic. We included a large population, with subjects managed during the first three waves. Therefore, we were able to consider patients with different features of the disease induced by following variants of the virus, which did not modify substantially the clinical presentation.

Beyond anamnestic characteristics, aged patients presented two relevant features: more severe respiratory failure than younger subjects, and more marked derangement of inflammatory markers. The difference between different age group was modest and, in all of them, respiratory parameters indicated a mild to moderate impairment, but it demonstrated to be an independent predictor of an adverse outcome [15] in youngest and oldest patients. Among youngest patients, upon admission, a very limited number, all in their forties and fifties, presented a respiratory impairment, possible sign of an early extensive lung involvement, and the association of this pattern with an unfavorable outcome was confirmed. On the other side, the presence of lung senescence posed elderly patients at high risk of evolving toward severe COVID-19, even in the presence of a mild impairment at presentation. Evaluating the response to the treatment, an element which proved to be a more powerful prognostic factor than the presence of the respiratory failure itself, could contribute to the early prognostic stratification in intermediate age groups.

An increased LDH level did not show an independent prognostic value. The biomarker was evaluated in several previous papers [16, 17], where it showed an association with increased mortality, but in this study population it was increased in the majority of patients, with a low discriminatory ability. Previous papers did not suggest to use different cut-offs and we tested this parameter, based on usual normal values. We performed a different choice for CRP, for which a relevant prognostic value was repeatedly confirmed, but always considering new cut-offs, derived from the median values of different populations, much higher than the normal value [8, 17, 18]. The range was really wide, and in the absence of an agreed value, we based our analysis on the median value of this population. We confirmed a significant association between an increased level of the biomarkers and an increased mortality only in elderly patients.

The lung function gradually declines after the age of 35 and modifications involve all cellular components, including the epithelium, pulmonary I mmune cells and the interstitium.

The respiratory epithelium, which represents the first line of defense against inhaled pathogens and other foreign material, shows prominent alterations with aging, with a reduced ciliary beat and mucociliary motility, due to a reduced secretion of surfactant in both upper and lower airways [19]. A reduction of the number of alveolar macrophages, the second-line of lung defense, as well as an impairment of their immune response, coupled with an increase in the number of neutrophils, was observed in the aging lung.

The pulmonary interstitium undergoes an increase in elastin degradation and senescence of fibroblasts [20]. A perturbation of the tissue architecture and impaired cell–cell communication are the final results of this component of the aging process.

The lung has to cope with a variety of exogenous stressors throughout the entire lifespan, so lung cellular lines developed several stress response pathways. The key element to guarantee an adequate defence is the balanced homeostasis, as under-responding may lead to inability to repair the insult, while over-responding can determine a damage to the lung itself. Unfortunately, several aspects of the aging process impacted on the ability of the lung cells to respond to exogenous noxae. All cell lines showed a reduced capacity to respond to oxidative stress [21] and a dysregulation in metabolic pathways in response to hypoxia. The final result is the increased susceptibility to respiratory tract infections, which represent the leading cause of death from any kind of infections in the elderly.

The detrimental rearrangement of the structure and function of the lung induced by the aging process is associated with “immunaging”, an age-dependent change of immune response [22]. Consistently with this mechanism, as well as with previous papers, we observed significantly higher values of inflammatory biomarkers, including CRP and neutrophil/lymphocyte ratio, with increasing age. The more marked elevation of CRP levels in the elderly is consistent with this pathophysiologic rearrangement and, in oldest patients, it showed an independent prognostic value, altogether with the presence of multiorgan dysfunction [23].

During the aging process, even in the absence of infections, there is an upregulation of the levels of pro-inflammatory cytokines, as well as a subclinical low-grade systemic pro-inflammatory state, known as “inflammaging” [24, 25]. The exact sources of inflammation that trigger inflammaging is unknown, but alveolar macrophages, which reside in the fluid lining alveolar epithelium, were thought to be involved as their count in the bronchoalveolar lavage from aged mice was higher compared to younger animals. The dysregulation of pulmonary innate immunity, as well as the alterations of adaptive component, with decreased lymphopoiesis, prevalence of myeloid production and diminished B cell function, increased lungs’ frailty and contributed to the increased susceptibility of the lung’s mucosal surface to airborne infections [26, 27].

With specific regard to SARS-COV-2 infections, the virus infects Type 2 alveolar epithelial cells, where the angiotensin-converting enzyme 2 (ACE2) represents the receptor for cellular entry [28]. A possible increased expression of the receptor with aging could enhance the viral entry into lung cells. In the same way, the increased neutrophilic infiltration and the alveolar macrophage activation, coupled with the background of inflammaging and the iper-inflammatory response caused by COVID-19, created a lethal combination with consequent worsening morbidity and mortality in the elderly population. In summary, all these mechanisms contributed to amplify a non-severe clinical picture upon ED entry, with a disproportionate increase in the fatality rate compared to young patients with a balanced immune system.

The retrospective and single center design represents significant limitations of the present study. In fact, these results may not be generalizable in the presence of different local admission and management policies. However, the use of a standardized model has limited the possibility of a subjective interpretation during data collection. We did not systematically collect data on body size and we were not able to include the presence of obesity among risk factors for an adverse outcome. In the same way, we did not collect systematically the data on the viral variants involved in COVID-19 infection, so we could not correlate the disease characteristics with specific variants. None of our patients was vaccinated at the time of the study, as in Italy the vaccination program began in January 2021 and, by May 2021, a large part of the population had not undergone the immunization.

Anyway, the availability of data about anamnestic characteristics, inflammatory activation and the presence of organ damage, by mean of parameters easily available upon ED admission, made these results applicable for the prognostic evaluation of patients at their very early in-hospital assessment.

## Conclusions

The presentation of COVID-19 was comparable in the different age groups. Aged patients presented a worse respiratory failure, even though always in the range of a mild to moderate impairment, and a more marked derangement of inflammatory biomarkers. In patients aged > 70 years, both alterations demonstrated an independent association with an increased in-hospital mortality. Therefore, upon the admission to the ED, in aged patients, even in the absence of a severe clinical picture, in-hospital mortality rate was high and an adequate disposition has to be planned.

## Data Availability

The datasets generated during and/or analyzed during the current study are available from the corresponding author on reasonable request.
